# Supplementary material for: Cervicovaginal-Microbiome Analysis by 16S Sequencing and Real-Time PCR in Patients from Novosibirsk (Russia) with Cervical Lesions and Several Years after Cancer Treatment
Source: Diagnostics (Basel). 2023 Jan 1;13(1):140. doi: 10.3390/diagnostics13010140 (PMC9818139; doi:10.3390/diagnostics13010140)
Supplement: Supplementary file 1 [file diagnostics-13-00140-s001.zip › diagnostics-2078730-supplementary.pdf]

Supplementary Materials

Cervicovaginal-microbiome analysis by 16S sequencing and real-time PCR in patients from Novosibirsk (Russia) with cervical lesions and several years after cancer treatment

Ivanov M.K. et al.

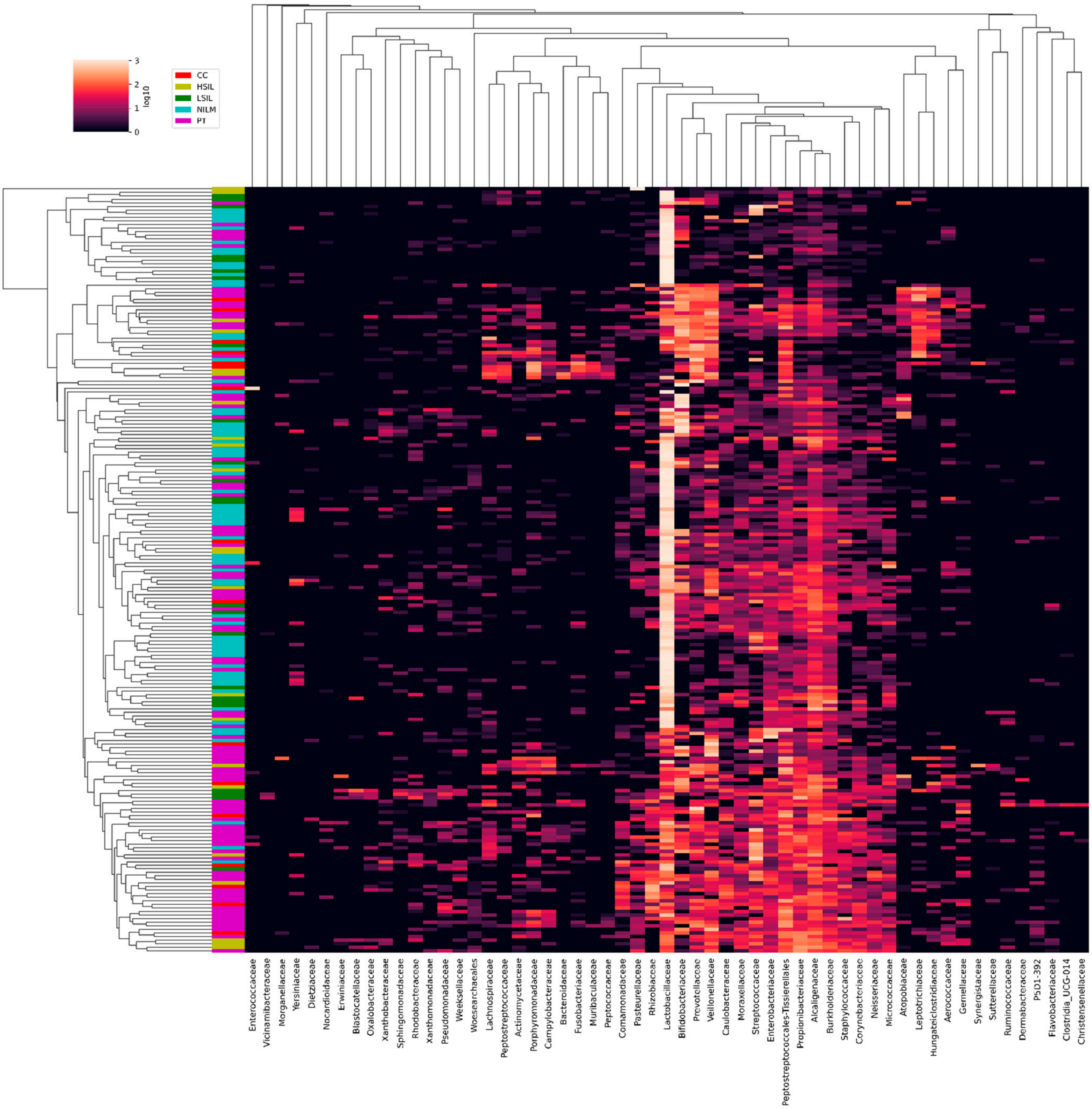

(a)

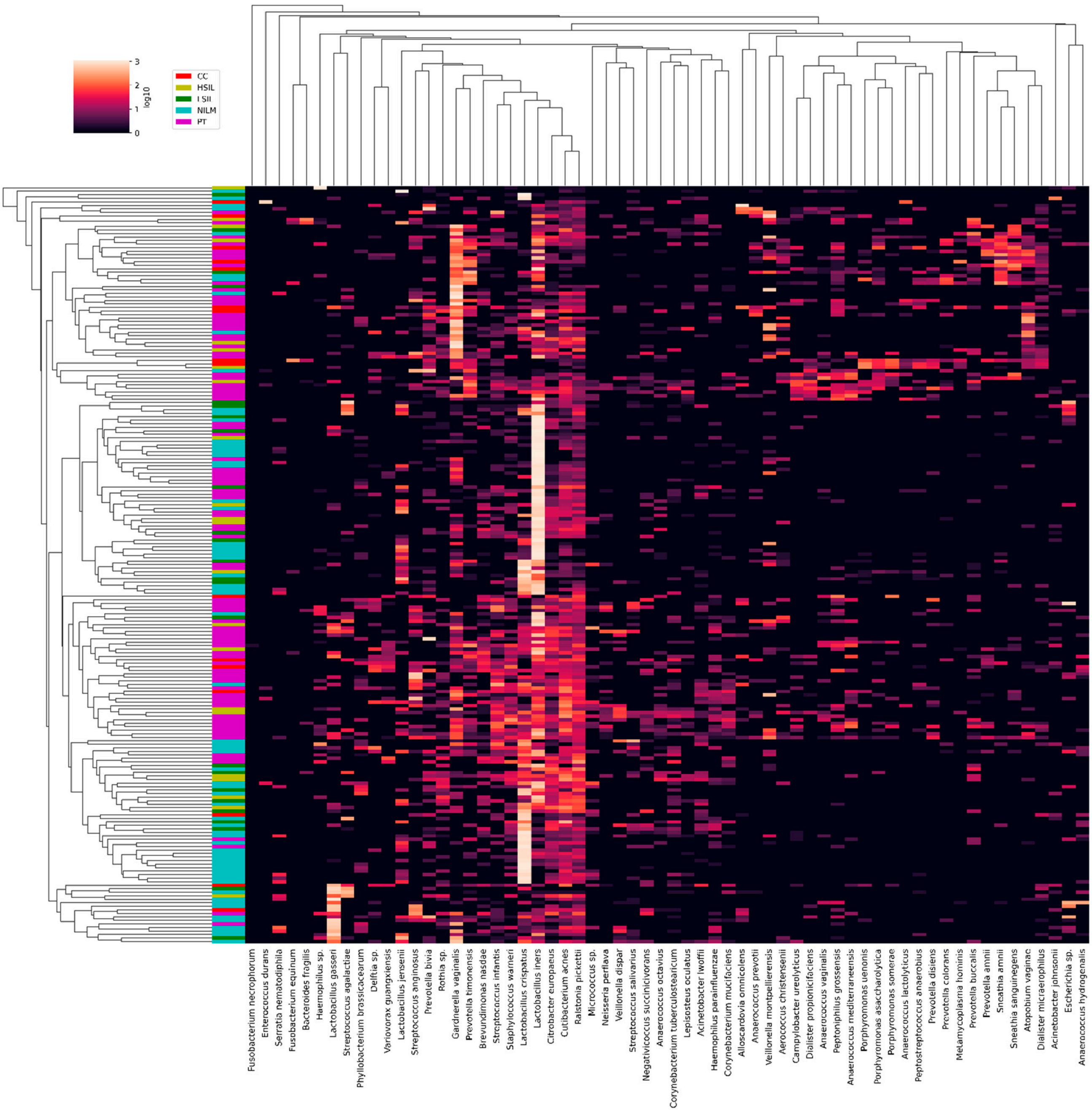

(b)

Figure S1. Community composition of cervical samples at the level of families (**a**) or species (**b**) as determined by massively parallel sequencing on the MiSeq platform. An unsupervised heatmap of the relative abundance of microbial taxa found in the cervicovaginal microbial communities of 234 patients: 72 NILMs (5 samples were excluded for technical reasons), 24 LSILs, 22 HSILs (2 samples were excluded for technical reasons), 15 CCs (1 sample was excluded for technical reasons), 101 PT samples, based on the Bray–Curtis dissimilarity metric. Diagnoses of patients are indicated by colors. (**a**) The families present in relative abundance of 1% in at least one sample are listed on the X axis. (**b**) Top 62 species (by abundance) are listed on the X axis. The cladograms at the top of the species' and families' names indicate the approximate evolutionary relationships between the species. LB species analysis by 16S metagenome sequencing. (**a**) The enrichment with lactobacilli (relative proportion of reads mapped to genomes of *Lactobacillus* spp.) at different diagnoses and post-treatment

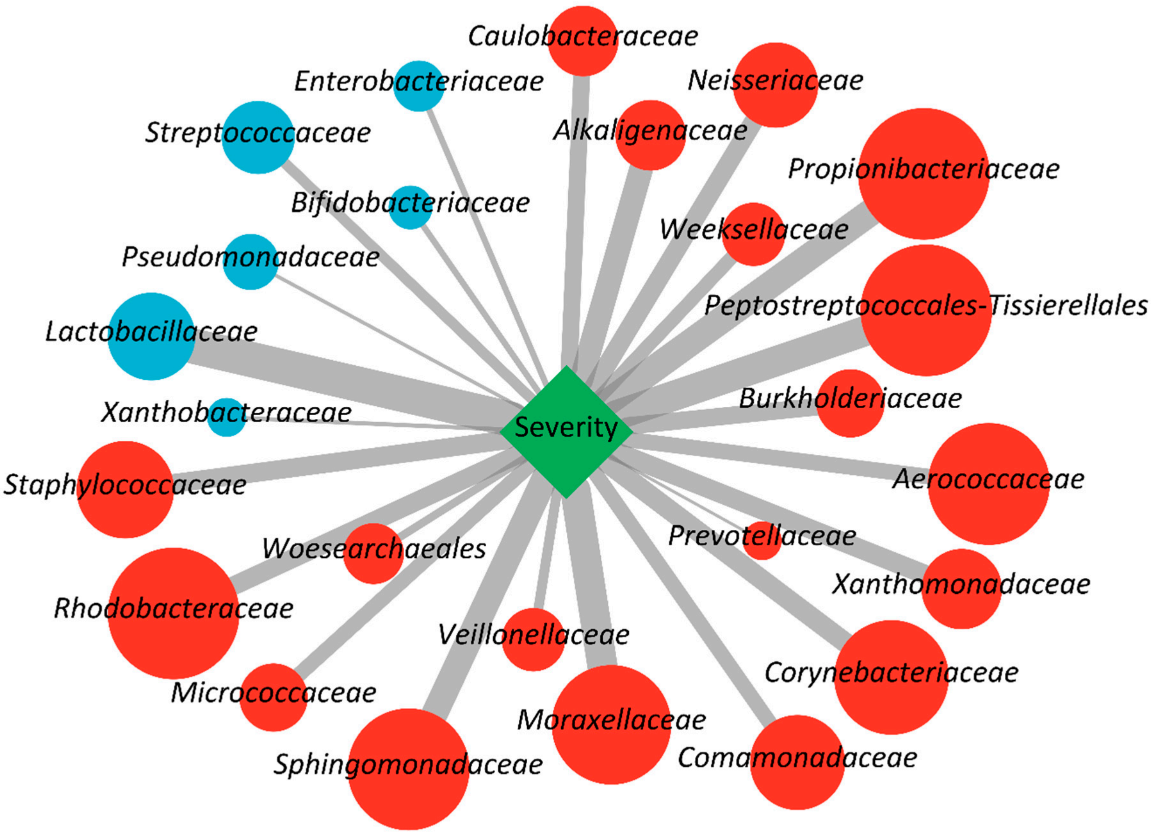

Figure S2. The correlation network between cervical lesion severity and microbiota at the family level. The nodes represent unique species, and the color of the nodes denotes an increase (red) or decrease (blue) in relative abundance when comparing patients with lesions and cancer and healthy controls. The size of the nodes shows the fold change values of the relative abundance in patients with high-grade lesions and cancer versus low-grade lesions healthy controls. The edges denote the correlation between the bacterial family and the lesion severity, the color of the edge represents a positive (red) or negative (blue) correlation, and the width denotes the strength of the correlation.

Table S1. Sequences of oligonucleotides used in the study. R6G: Rhodamine 6G (Rhodamine 590); ROX: Rhodamine X (carboxy-X-rhodamine); BHQ1: Black Hole Quencher-1; BHQ1: Black Hole Quencher-2; LNA: locked nucleic acid; R: reverse; F: forward.

ID	Type	Sequence (5′ → 3′)	Purpose
HMBS-R	Forward PCR primer	GTGGCTACTGTCTGATGTAGAA	Human HMBS gene amplification
HMBS-R	Reverse PCR primer	GGTCTCGAACTTGTGATCCT	Human HMBS gene amplification
HMBS-P	TaqMan PCR probe	(R6G)-TCACGCCTG(T-BHQ1)AATCCCAGCACTTTGGGA-p	Human HMBS gene amplification
BVcF2	Forward PCR primer	AACAGGATTAGATACCCTGGTA	Pan-bacterial 16S rRNA gene amplification
BVcR2	Reverse PCR primer	CATCTCACGACACGAGCT	Pan-bacterial 16S rRNA gene amplification
BVpZ2	TaqMan PCR probe	(ROX)- GCTAAG (C-LNA)GAAAG (C-LNA)ATTAAGCATCCCACCTG-BHQ2	Pan-bacterial 16S rRNA gene amplification
Fus342F	Forward PCR primer	ACACTCTTTCCCTACACGACGCTCTTCCGATCTCTACGGGAGGCAGCAG	16S Library Preparation PCR Round 1
Fus806R	Reverse PCR primer	GTGACTGGAGTTCAGACGTGTGCTCTTCCGATCTGGACTACCGGGGTATCT	16S Library Preparation PCR Round 1
P7i	Indexed PCR primer	CAAGCAGAAGACGGCATACGAGAT-[Nextera-8nt-index]-GTGACTGGAGTTCAGACGTGTGCTCTTCCGATC*T	16S Library Preparation PCR Round 2
P5i	Indexed PCR primer	AATGATACGGCGACCACCGAGATCTACAC-[Nextera-8nt-index]-ACACTCTTTCCCTACACGACGCTCTTCCGATC*T	16S Library Preparation PCR Round 2
ilP-f	Forward PCR primer	ATGATACGGCGACCACC	Quantification of libraries
ilP-r	Reverse PCR primer	CAAGCAGAAGACGGCATAC	Quantification of libraries
ilP-p	TaqMan PCR probe	(ROX)-ACACTCTT[T-BHQ2]CCCTACACGACGCTCT-p	Quantification of libraries

Table S2. P-values for pairwise comparisons of:

a) CVM α-diversity parameters at different diagnoses and post-treatment (see Figure 2); CRT, chemoradiotherapy; RT, radiotherapy; ST, surgical treatment; CT, combined RT and ST; PT, post-treatment group; PT+, patients undergoing surgery (as the main method or as part of combined regimens); PT-, patients without such an intervention;
b) relative proportion of reads mapped to genomes of *Lactobacillus* spp. at different diagnoses and post-treatment, as determined by 16S rRNA gene sequencing (see Figure 3(a));
c) relative abundance counts of *Peptococcales-Tissierellales*, *Bifidobacteriaceae*, *Veillonellaceae,* and *Sphingomonadaceae* at different diagnoses and post-treatment, as determined by 16S rRNA gene sequencing (see Figure 4a);
d) relative abundance counts of *Propionibacteriaceae*, *Alcaligenaceae*, *Burkholderiaceae,* and *Moraxellaceae* at different diagnoses and post-treatment, as determined by 16S rRNA gene sequencing (see Figure 4b);
e) relative abundance counts of *Cutibacterium acnes* at different diagnoses and post-treatment, as determined by 16S rRNA gene sequencing (see Figure 4d);
f) enrichment with *Lactobacillus iners*, non-*iners* lactobacilli, *Gardnerella vaginalis*, and *Prevotella* species at different diagnoses and in post-treatment subgroups, as determined by 16S rRNA gene sequencing (see Figure 5d).
g) relative abundance of lactobacilli, common bacterial-vaginosis–associated anaerobes (*Gardnerella vaginalis*, *Atopobium vaginae*, *Prevotella* spp., and the *Leptotrichia amnionii* group summarized) and common aerobic-vaginitis–associated aerobes (*Streptococcus* spp., *Staphylococcus* spp., and *Enterococcus* spp. summarized) as assessed by the BioFlor kit at different diagnoses and in different post-treatment subgroups (see Figure 6c).

a)

**Faith phylogenetic diversity**	**NILM vs HSIL**	0.012722
	**NILM vs CC**	0.000344
	**NILM vs PT-**	4.78E-07
**OTUs**	**NILM vs HSIL**	0.017173
	**NILM vs CC**	7.44E-05
	**NILM vs PT-**	7.6E-08
**Shannon index**	**NILM vs HSIL**	0.000631
	**NILM vs CC**	8.79E-09
	**NILM vs PT-**	2.4E-07

b)

**NILM vs HSIL**	0.002897
**NILM vs CC**	0.000001
**NILM vs PT**	0.000000
**LSIL vs CC**	0.000315
**LSIL vs PT**	0.004110
**HSIL vs CC**	0.032764
**CC vs PT**	0.013019

c)

Family	NILM vs CC	LSIL vs PT	LSIL vs CC
Peptostreptococcales-Tissierellales	8.78E-06	0.00465	0.013925472
Bifidobacteriaceae	>0.05	0.030166	>0.05
Veillonellaceae	>0.05	>0.05	>0.05
Sphingomonadaceae	0.000718	0.000769	>0.05

d)

Family	NILM vs CC	HSIL vs PT	CC vs PT
Proponibacteriaceae	0.0014069	>0.05	0.038993246
Alkaligenaceae	0.0006585	0.017628	0.000497009
Burkholderiaceae	>0.05	>0.05	>0.05
Moraxellaceae	9.512E-05	>0.05	6.26604E-06

e)

**NILM vs CC**	0.0003317
**CC vs ST**	0.0409828
**CC vs CT**	0.020345

f)

	**NILM vs CC**	0.0004	(***)
	**NILM vs RT**	0.0000	(***)
Lactobacillus spp. non-*iners*	**LSIL vs RT**	0.0280	(*)
	**LSIL vs CC**	0.0395	(*)
	**CC vs RT**	0.0061	(**)
	**CC vs ST**	0.0358	(*)
Lactobacillus iners	**CC vs CT**	0.0313	(*)

g)

	**NILM vs**					
	**HSIL**	**CC**	**CRT**	**RT**	**ST**	**CT**
Lactobacillus	0.046043842	1.59E-05	3.21E-05	1.1E-08	>0.05	>0.05
Anaerobes	0.043838567	0.008897	0.000202	4.19E-09	0.000726131	0.036949
Aerobes	>0.05	>0.05	0.004397	0.000112	>0.05	>0.05
	**LSIL vs**					
	**HSIL**	**CC**	**CRT**	**RT**	**ST**	**CT**
Lactobacillus	>0.05	0.007181486	>0.05	0.006903	>0.05	>0.05
Anaerobes	>0.05	>0.05	>0.05	>0.05	>0.05	>0.05
Aerobes	>0.05	>0.05	>0.05	>0.05	>0.05	>0.05
